# Effects of Different Fertilization Patterns on the Content of Quality Indices and the Formation of Aroma Characteristics of Tea Leaves

**DOI:** 10.3390/foods14183194

**Published:** 2025-09-13

**Authors:** Qi Zhang, Songhan Guo, Yulin Wang, Yangxin Luo, Yankun Liao, Xiaoli Jia, Bitong Zhu, Jianghua Ye, Haibin Wang

**Affiliations:** 1 College of Tea and Food Science, Wuyi University, Wuyishan 354300, China; zhangqi1113@wuyiu.edu.cn (Q.Z.);; 2 College of Life Science, Longyan University, Longyan 364012, China; 3 College of Life Sciences, Fujian Normal University, Fuzhou 350002, China

**Keywords:** *Camellia sinensis*, fertilizer application, volatile components, aromatic characteristics, quality

## Abstract

Fertilization is a necessary measure in the cultivation and management of the tea bush, and the use of different fertilizers significantly affects the quality of tea leaves. In this study, 100% chemical fertilizer (CF), 100% organic fertilizer (OF), and 50% chemical fertilizer + 50% organic fertilizer (COF) were used to analyze the effects of different fertilizer treatments on the quality indices and volatile component compositions of tea leaves. The results showed that COF treatment significantly increased the content of water extract, tea polyphenols, theanine, free amino acids, and soluble sugar, but decreased the content of total flavonoids and caffeine, indicating that COF treatment has an advantage in improving the overall quality of tea leaves. Analysis of volatile components showed that different fertilizer treatments significantly affected the content of eleven characteristic metabolites in tea bush leaves, of which four characteristic metabolites were most abundant under CF treatment, eight under OF treatment, and only one under COF treatment. The odor characteristics and their intensity analyses showed that the 11 characteristic volatile components mainly exhibited three types of odor characteristics, such as woody, green, and floral, of which OF treatment enhanced woody and green intensities, while CF treatment enhanced floral intensity. Taken together, different fertilizer treatments could regulate the quality indices and volatile components of tea leaves, while the use of chemical fertilizers or organic fertilizers alone was conducive to enhancing the intensity of individual special odor characteristics in tea leaves. Overall, the combination of chemical fertilizers and organic fertilizers was more conducive to the improvement of tea quality. This study provides a theoretical basis for optimizing the fertilization strategy of tea bushes to improve tea quality.

## 1. Introduction

As one of the most important cash crops in the world, the quality of the tea bush (*Camellia sinensis*) directly affects the market value and consumer acceptance of tea [1]. With the continuous increase in global tea consumption demand and consumers’ pursuit of high-quality tea, how to improve tea quality through scientific cultivation and management has become an important issue in tea research [2]. Fertilization, as a core part of tea plantation management, not only affects the growth and development of tea bushes, but also directly regulates the synthesis and accumulation of various flavor substances and functional components in tea [3,4]. In recent years, due to the in-depth development of eco-agriculture concepts and the proposal of “two-carbon” strategic goals the fertilization modes of tea plantations are undergoing a shift from the pursuit of yield to quality and ecology, which makes an in-depth study of the influence mechanism of different fertilizer treatments on the quality of tea leaves of great theoretical significance and practical value [5].

The quality evaluation of tea leaves mainly analyzes their biochemical quality indicators and aroma component contents. Cultivation measures have a significant impact on the content of tea quality components, and fertilizer use is a necessary measure in the cultivation and management of tea trees [6]. Although traditional chemical fertilizers can quickly replenish soil nutrients, their single long-term use has revealed many problems, including soil acidification, microbial community imbalance, etc., which can negatively affect tea quality [7,8]. Organic fertilizers (e.g., animal-based organic fertilizers, mineral-based organic fertilizers, plant-based organic fertilizers) have attracted a lot of attention because of their ability to improve soil structure and microbial activity, but their slow release of nutrients cannot quickly meet the growth demands of tea bushes [9,10]. In recent years, there has been a growing body of research investigating how fertilizer application strategies affect tea quality. For instance, Xi et al. [11] observed that elevated potassium fertilizer application not only stimulates tea bush growth, thereby increasing yield, but also exerts a positive effect on tea quality, with a particular enhancement in tea polyphenol content. Ye et al. [12] demonstrated that the application of sheep manure organic fertilizer to acidified tea plantation soils can effectively boost both tea yield and quality. Conversely, Piyasena & Hettiarachchi [13] found that the exclusive use of organic fertilizers compared to chemical fertilizers led to a reduction in the free amino acid content of tea leaves, with theanine being particularly affected. Additionally, Liu et al. [14] evaluated the influence of three types of organic fertilizers on tea yield and quality, and found that compared to chemical fertilizers, bio-organic fertilizers provided the most significant improvement in both yield and quality. It can be seen that organic fertilizers do have a certain effect on the improvement of tea quality, but there are still deficiencies. Furthermore, a large number of studies only use the conventional tea physiological and biochemical quality indices to evaluate tea quality, and there are still some defects. In this context, the combination of organic fertilizers and chemical fertilizers as a compromise can theoretically take into account the balance of fast-acting and long-lasting, yield and quality, and the impact on tea quality. However, the volatile components of the mechanism have not been systematically studied [15]. As a key component in measuring tea aroma quality, the types and contents of volatile components are regulated by a variety of factors, of which fertilization management is considered to be one of the most important influencing factors [16,17]. However, studies on how different fertilization patterns affect the synthesis of characteristic aroma substances by altering the metabolic pathways of tea bushes are still insufficient, limiting the ability of targeted regulation of tea aroma quality through fertilization management.

Tea quality formation is a complicated physiological and biochemical process involving synergistic changes in multiple primary and secondary metabolites [18]. From a metabolic point of view, fertilization treatments may affect tea quality through a variety of pathways: the first is to change the effectiveness of nutrients, thus affecting the uptake and utilization of mineral elements by tea bushes [19]; the second is to regulate the structure of soil microbial communities, which indirectly affects the metabolic activities of tea bush roots [20]; and the third is to directly participate in controlling various metabolic pathways in the body of tea bushes, such as carbon and nitrogen metabolism, benzylpropane metabolism, terpene synthesis, and other key pathways [21]. These metabolic changes are ultimately reflected in various quality indices, including the content of basic components such as tea polyphenols, caffeine, and amino acids, as well as the composition and proportion of various volatile components [22]. Notably, the responses of different quality indices to fertilization treatments may differ or even be conflicting, requiring a systematic and comprehensive approach when evaluating the effects of fertilization. However, there are still limited studies that can simultaneously examine the influences of fertilization on multiple tea bush quality indices, which to some extent hinders accurate assessment of the comprehensive benefits of different fertilization patterns.

Accordingly, this study used three kinds of fertilization patterns, including 100% chemical fertilizer (CF), 50% chemical fertilizer + 50% organic fertilizer (COF), and 100% organic fertilizer (OF), to treat tea bushes; analyze the effects of different fertilizer treatments on the conventional quality indices of tea bush leaves on the one hand, and analyze the effects of different fertilizer treatments on the composition and content of volatile components in tea bush leaves on the other hand; and screen for characteristic volatile components significantly affected by fertilization treatments and analyze their odor characteristics. The study aims to systematically evaluate the comprehensive effects of different fertilizer treatments on the quality indices and volatile components of tea leaves, provide a theoretical basis and technical support for the scientific fertilization of tea plantations to improve tea quality, and provide an important reference for the establishment of a high-quality and high-efficiency tea plantation management system.

## 2. Materials and Methods

### 2.1. Basic Information of the Experimental Site

This research was conducted in Xibian Village, Xingcun Town, Wuyishan City (Nanping, Fujian province), situated at the geographical coordinates 27°39′43.3728″ N, 117°53′33.5796″ E and at an elevation of 228 m. The study area falls within the humid central subtropical monsoon climate zone, characterized by a mean annual temperature of 12 °C and annual rainfall averaging 1284 mm. Precipitation is concentrated primarily between April and June, with heavy seasonal rains creating favorable conditions for tea cultivation. The tea bushes under investigation were 14 years old and grown in acid red soil with a pH of 4.21. Additional soil properties included the following: organic matter (2.07 g/kg), total nitrogen (6.75 g/kg), total phosphorus (0.79 g/kg), total potassium (1.62 g/kg), available nitrogen (2.01 mg/kg), available phosphorus (4.41 mg/kg), and available potassium (127.97 mg/kg).

### 2.2. Experimental Design and Sample Sampling

The tea bush variety at the experimental site was Jinguanyin (*Camellia sinensis*). The study was designed to treat tea bushes with three different types of fertilizers, such as 100% chemical fertilizer (CF), 50% chemical fertilizer + 50% organic fertilizer (COF), and 100% organic fertilizer (OF). Three independently replicated experimental areas were set up for each treatment, each with an area of 100 m^2^. The selected chemical fertilizers were compound fertilizers (15% N, 15% P_2_O_5_, 15% K_2_O), with a recommended application rate of 0.07 kg/m^2^; and the selected organic fertilizers were plant humus organic fertilizer (3.5% N, 3% P_2_O_5_, 3% K_2_O, and 45% organic matter), purchased from China Zhongnong Lvkang (Beijing, China) Biotechnology Co. Ltd., and the recommended application rate was 0.30 kg/m^2^. Therefore, the CF treatment was the use of chemical fertilizer only, with an application rate of 0.07 kg/m^2^; the OF treatment was the use of organic fertilizer only, with an application rate of 0.30 kg/m^2^; and the COF was the combined application of chemical and organic fertilizers, with an application rate of 0.035 kg/m^2^ chemical fertilizer + 0.15 kg/m^2^ organic fertilizer. According to the traditional fertilization methods used in the cultivation of Wuyi rock tea, the specific time and method of fertilizer application were as follows: in October 2024, a trench was dug 30 cm from the main stem of the tea bush, 20 cm wide and 25 cm deep, and the fertilizer was evenly spread according to the experimental design at one time, and then covered with soil. In May 2025, tea bush leaves (two leaves and one core) were collected, and parts of them were used for the measurement of the conventional quality indices, and parts of them were used for the measurement of the volatile components. The specific sampling method was as follows: under different fertilizer treatments, 6 tea bushes were randomly selected in each test area, and the two leaves and one core were picked, mixed, and immediately placed in an ice box as a replication. Each fertilization treatment was set for three independent replicates.

### 2.3. Measurement of Tea Quality Indices

Tea samples were first placed at 105 °C for 2 h, then dried at 80 °C until constant weight. After cooling, the samples were ground and sieved through a 2 mm sieve, then used for quality index measurements. The quality indices primarily measured were water extract, tea polyphenols, theanine, caffeine, total flavonoids, free amino acids, and soluble sugar, with specific methods referenced from Wang et al. [23]. Water extract was prepared by reflux extraction with boiling water, and the filtrate was dried and analyzed. Tea polyphenols were measured utilizing the Folin–Ciocalteu colorimetric method. Specifically, the extract was mixed with 1.5 mL of 10% Folin’s reagent and reacted for 5 min, then 4 mL of 7.5% Na_2_CO_3_ solution was added, and the mixture was then reacted in the dark for 60 min. The absorbance was measured at 765 nm, and quantification was performed utilizing a gallic acid standard curve. Caffeine was measured utilizing the lead acetate–alkali UV spectrophotometric method. The extract was mixed with 2 mL of 10% lead acetate–alkali solution to precipitate interferents, filtered, and then the absorbance was measured at 276 nm utilizing a caffeine standard curve for quantification. Theanine was measured utilizing high-performance liquid chromatography (HPLC). An Agilent 1100 Series HPLC system equipped with a Discovery^®^ C18 column (25 cm × 4.6 mm, 5 μm) was used. Mobile phase A was 0.1% trifluoroacetic acid and mobile phase B was acetonitrile. For gradient elution (0–10 min: 95% A → 85% A), a flow rate of 1.0 mL/min, column temperature of 30 °C, and detection wavelength of 210 nm were used. Quantification was performed utilizing a theanine standard curve. Total flavonoids were measured utilizing the rutin colorimetric method, i.e., the extract was reacted with 0.3 mL of 5% NaNO_2_ for 6 min, followed by the addition of 0.3 mL of 10% AlCl_3_ and reacted for another 6 min, then 4 mL of 1 M NaOH was added, and the absorbance was measured at 510 nm, with quantification based on the rutin standard curve. Free amino acids were measured utilizing an automatic amino acid analyzer (L-8900, Hitachi High Tech, Tokyo, Japan). Soluble sugars were measured utilizing the anthrone colorimetric method, where the extract was mixed with 0.2% anthrone-concentrated sulfuric acid reagent, reacted in a boiling water bath for 10 min, and then the absorbance was measured at 625 nm, with quantification utilizing a glucose standard curve.

### 2.4. Determination of Volatile Components Content in Tea Leaves

Volatile tea components were analyzed by Head Space-Solid Phase Microextraction (HS-SPME) coupled with gas chromatography mass spectrometry (GC-MS; Agilent 8890B GC system and 7000D MS detector, Palo Alto, CA, USA) [24]. For sample preparation, 2 g of ground tea was weighed into a 20 mL headspace vial. A 5 μL aliquot of ethyl decanoate (50 μg/mL) was introduced as an internal standard via the vial’s liner tube before sealing. The sealed vial was incubated at 50 °C for 30 min to facilitate complete volatilization of aromatic compounds. Subsequently, a SPME fiber (50/30 m, DVB/CAR/PDMS, Supelco, Bellefonte, PA, USA) was exposed to the headspace for 30 min, then immediately desorbed in the GC-MS injection port for 5 min. Chromatographic separation was performed on an HP-5MS column (Agilent, 30 m × 0.25 mm, 0.25 µm). The temperature program was as follows: initial hold at 50 °C for 2 min ramp to 80 °C at 2 °C/min (4 min hold), increase to 180 °C at 5 °C/min (5 min hold), and final elevation to 220 °C at 10 °C/min (10 min hold). High-purity helium served as the carrier gas at a constant flow rate of 1.0 mL/min under splitless injection conditions. Mass spectrometric detection employed electron ionization (EI) in full-scan mode (*m*/*z* 40–600), with ion source, quadrupole, and transfer line temperatures maintained at 230 °C, 150 °C, and 280 °C, respectively. The qualitative analysis of the compounds was performed by comparing the characteristic ions of each compound with the NIST 20 mass spectrometry database after subtracting the background. Quantitative analysis was performed by calculating the peak area of the characteristic ions after integration and correction [25].

### 2.5. Statistical Analysis

The raw data were initially organized utilizing Microsoft Office 2021, and then analyzed and visually mapped utilizing Rstudio software (v 4.2.3). The ANOVA method was used to analyze the differences between different indices. Quality indices and volatile metabolite content were analyzed and mapped utilizing the R package gghalves 0.1.4. Venn and stacked plots of volatile compound compositions were produced utilizing the R packages ggvenn 0.1.10 and ggplot2 3.5.2. Heat maps of volatile compound trends were produced utilizing the R packages pheatmap 1.0.13 and vegan 2.7.1. The R package for the ANOVA difference test for volatile components was stats 4.4.2. The R packages for OPLS-DA model construction for CF, COF, and OF were ropls and mixOmics. R packages for bubble feature map screening and TOPSIS analysis for volatile components were ggplot2 3.4.0 and dplyr 1.1.4. The R package used for odor wheel production was circlize version 0.4.15.

## 3. Results

### 3.1. Effects of Different Fertilizer Treatments on Quality Indices of Tea Bush Leaves

**Figure 1:**
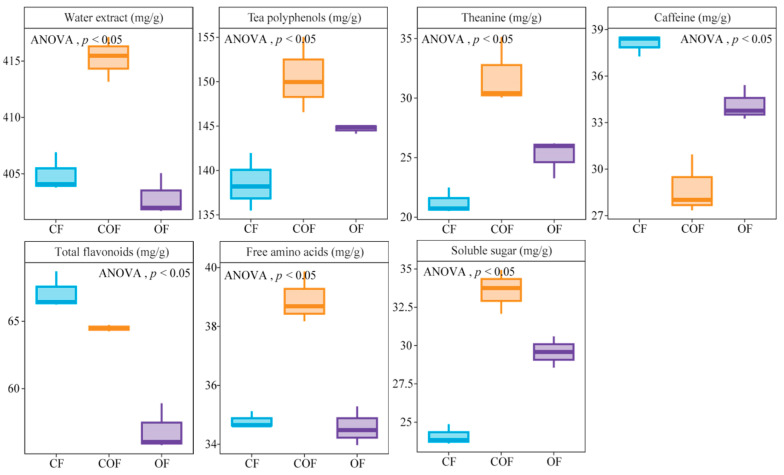
Effect of different fertilizer treatments on the content of quality indices of tea bush leaves. Note: CF, COF, and OF denote 100% chemical fertilizer treatment, 50% chemical fertilizer + 50% organic fertilizer treatment, and 100% organic fertilizer treatment, respectively.

As illustrated in Figure 1, analysis of tea leaf quality indices across fertilizer treatments revealed distinct patterns. COF treatment exhibited the highest content of water extract (415.26 mg/g), tea polyphenols (150.53 mg/g), theanine (31.87 mg/g), free amino acids (38.91 mg/g), and soluble sugar (33.58 mg/g), alongside the lowest caffeine content (28.77 mg/g). In contrast, CF treatment yielded the maximum levels of caffeine (38.07 mg/g) and total flavonoids (67.13 mg/g), but showed the lowest contents for tea polyphenols (138.56 mg/g), theanine (21.25 mg/g), and soluble sugar (24.11 mg/g). Under OF treatment, water extract (402.93 mg/g), total flavonoids (56.91 mg/g), and free amino acids (34.58 mg/g) were the lowest among all groups, while other quality indices were at intermediate levels between COF and CF treatments. It is evident that COF treatment most effectively enhanced the accumulation of major quality indices in tea leaves.

### 3.2. Effect of Different Fertilizer Treatments on the Composition of Volatile Components in Tea Bush Leaves

**Figure 2:**
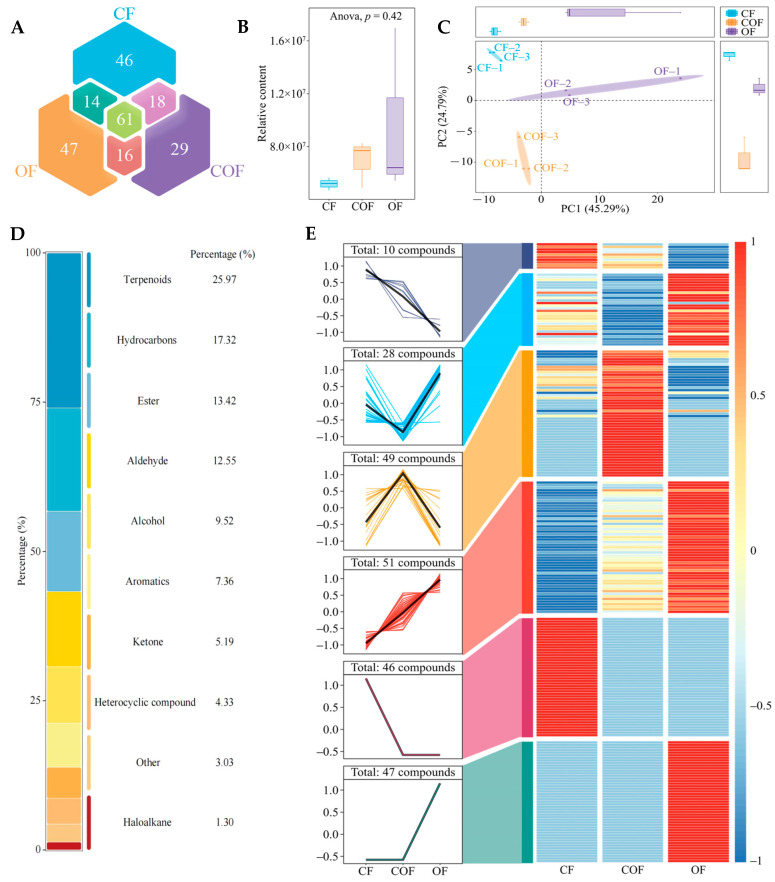
Effect of different fertilizer treatments on volatile compound composition of tea bush leaves. Note: CF, COF, and OF denote 100% chemical fertilizer treatment, 50% chemical fertilizer + 50% organic fertilizer treatment, and 100% organic fertilizer treatment, respectively. (**A**) Venn diagram analysis of volatile compound composition of tea bush leaves; (**B**) analysis of the total content of volatile components of tea bush leaves; (**C**) principal component analysis of volatile compound content of tea bush leaves after different fertilizer treatments (**D**): analysis of the classification of volatile components and their quantitative percentage; (**E**) trend analysis of volatile components content.

Analysis of tea leaf volatile components across fertilizer treatments (Figure 2A) identified 231 distinct volatile components in total. Among these, 61 compounds were common to CF, COF, and OF treatments, while 46, 29, and 47 unique compounds were exclusive to CF, COF, and OF, respectively. Further assessment of total volatile content (Figure 2B) showed no statistically significant differences between treatments (*p* = 0.42). Principal component analysis (PCA) based on volatile compound contents (Figure 2C) revealed that variations existed in volatile compound contents across fertilizer treatments. The two principal components collectively explained 70.08% of the variance, enabling clear discrimination between CF, COF, and OF. To explore compositional differences, detected volatiles were categorized into 10 chemical classes (Figure 2D). Terpenoids (25.97%), hydrocarbons (17.32%), and esters (13.42%) emerged as the three most abundant classes, accounting for the largest proportion of total volatile content. The results of the trend analysis of volatile component contents (Figure 2E) indicated that six main trends existed in the contents of 231 volatile components under different fertilizer treatments, of which, 10 volatile components presented CF > COF > OF, 28 volatile components presented OF > CF > COF, 49 volatile components presented COF > CF > OF, 51 volatile components presented OF > COF > CF, 46 volatile components presented CF > COF = OF, and 47 volatile components presented OF > COF = CF. It is evident that the content of different volatile components in tea bush leaves altered significantly under different fertilizer treatments.

### 3.3. Screening of Tea Bush Leaves for Differentially Characteristic Volatile Components After Different Fertilizer Treatments

**Figure 3:**
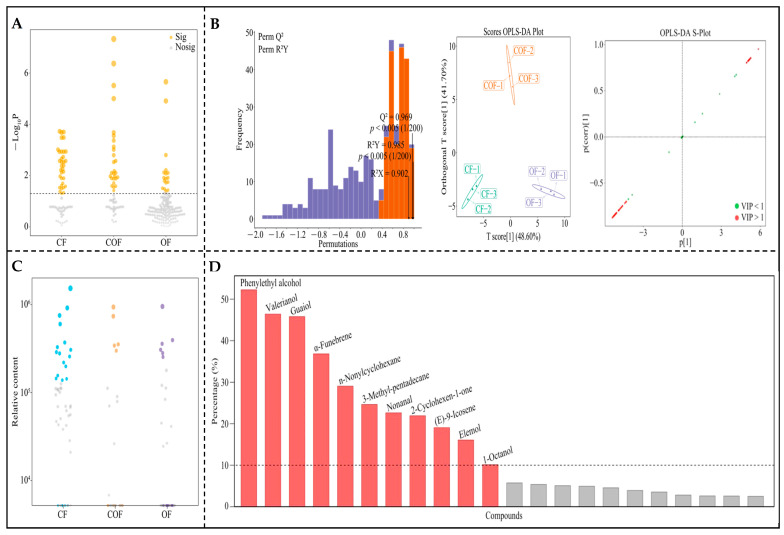
Screening of characteristic volatile components that significantly changed in tea bush leaves after different fertilizer treatments. Note: CF, COF, and OF denote 100% chemical fertilizer treatment, 50% chemical fertilizer + 50% organic fertilizer treatment, and 100% organic fertilizer treatment, respectively. (**A**) ANOVA test to screen for volatile components that significantly changed after different fertilizer treatments; (**B**) OPLS-DA model construction of CF, COF, and OF to screen for key differential volatile components; (**C**) bubble feature map to screen for characteristic volatile components that significantly changed; (**D**) TOPSIS analysis of the contribution of characteristic volatile components in different fertilizer treatments.

**Figure 4:**
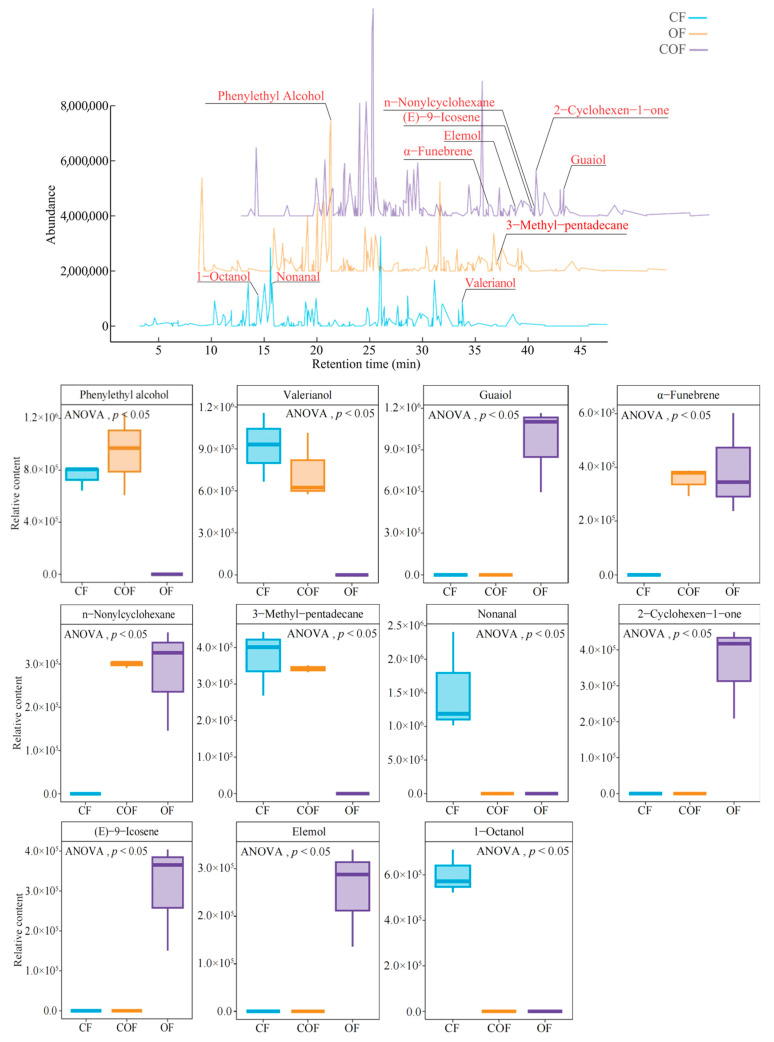
Effects of different fertilizer treatments on the content of characteristic volatile components of tea bush leaves. Note: CF, COF, and OF denote 100% chemical fertilizer treatment, 50% chemical fertilizer + 50% organic fertilizer treatment, and 100% organic fertilizer treatment, respectively.

From the above analysis, the present study continued to screen for characteristic volatile components that were significantly different in tea bush leaves under different fertilizer treatments. First, these volatile components from different fertilizer treatments were used for the ANOVA test, and it was found (Figure 3A) that among CF, COF, and OF, 78 volatile components showed significant differences. The OPLS-DA model of CF, COF, and OF was constructed with 78 significantly different volatile components to screen key different volatile components, and the results showed (Figure 3B) that the fit (R^2^Y) and predictive (Q^2^) elements of the model reached the significance level (*p* < 0.005), and the model could effectively differentiate CF, COF, and OF. Finally, 53 key volatile components (VIP > 1) were derived from the S-plot diagrams of the model, and CF, COF, and OF could be distinguished. Further, the bubble feature map revealed (Figure 3C) that 22 characteristic volatile components could significantly distinguish CF, COF, and OF. TOPSIS analysis based on 22 characteristic volatile components found (Figure 3D) that only 11 characteristic volatile components contributed more than 10% to distinguishing CF, COF, and OF. Further analysis of the contents of the 11 characteristic volatile components found (Figure 4) that significant differences existed in their contents under different fertilizer treatments. Among them, valerianol, 3-methyl-pentadecane, nonanal, and 1-octanol had the the highest content under the CF treatment, phenylethyl alcohol had the highest content under the COF treatment, and guaiol, α-funebrene, n-nonylcyclohexane, 2-cyclohexen-1-one, (E)-9-icosene, and elemol had the highest content under OF treatment. It is evident that different fertilizer treatments significantly affected the level of volatile components in tea bush leaves, particularly 11 characteristic volatile components, and the pattern of the effect of different fertilizer treatments on the level of characteristic volatile components differed significantly.

### 3.4. Odor Characteristics of Characteristic Volatile Components and Their Intensity Analysis

**Figure 5:**
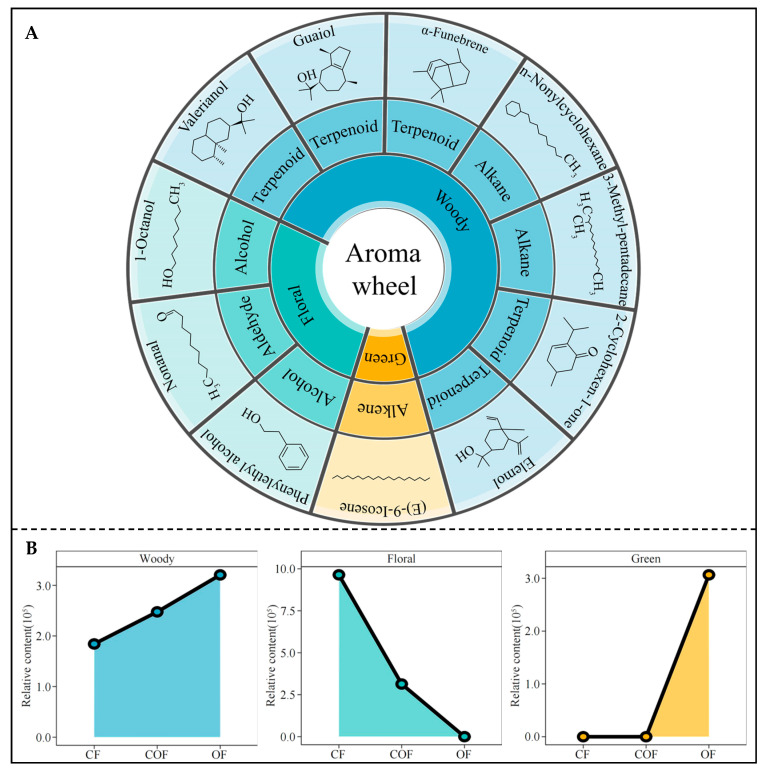
Effects of different fertilizer treatments on odor characteristics of tea bush leaves. Note: CF, COF, and OF denote 100% chemical fertilizer treatment, 50% chemical fertilizer + 50% organic fertilizer treatment, and 100% organic fertilizer treatment, respectively. (**A**) Odor characteristics of different characteristic volatile components; (**B**) effects of different fertilizer treatments on odor characteristics of tea bush leaves.

From the above-mentioned analysis, the present study continued to analyze the odor characteristics of the 11 characteristic volatile components, and the results showed (Figure 5A) that the characteristic volatile components mainly presented three kinds of odor characteristics, such as woody, green, and floral. Among them, valerianol, guaiol, α-funebrene. n-nonylcyclohexane, 3-methyl-pentadecane, 2-cyclohexen-1-one, and elemol mainly presented the woody odor characteristics; (E)-9-icosene mainly presented the green odor characteristics; and phenylethyl alcohol, nonanal, and 1-octanol predominantly presented the floral odor characteristics. Further analysis of the intensity of the three odor characteristics presented by the 11 characteristic volatile components revealed (Figure 5B) that the intensity of woody was OF > COF > CF, the intensity of floral was CF > COF > OF, and the intensity of green was OF > COF = CF. It is clear that OF treatment was beneficial to enhance the intensity of odor characteristics such as woody and green in tea leaves, while CF treatment was beneficial to enhance the intensity of floral, and the effect of COF treatment on the intensity of the three odor characteristics was intermediate.

## 4. Discussion

In recent years, with a growing emphasis on eco-agriculture and low-carbon cultivation there has been a growing academic focus on how fertilization patterns affect tea quality in plantations [26]. This study examined the effects of different fertilizer treatments on tea quality indices and found that, compared with CF and OF, COF was more conducive to increasing the content of most tea quality indices, except for caffeine and total flavonoids. Chemical fertilizers dissolved quickly, allowing rapid nutrient uptake by tea bushes to boost yield and quality [27]. However, long-term application can cause soil acidification and deterioration of texture, which in turn disrupts tea bush nutrient metabolism, reduces quality-related compounds, and impairs flavor [28,29]. Organic fertilizers, although beneficial for improving soil structure, enhancing microbial diversity, and maintaining soil health [12], have low nutrient content and slow release rates, failing to meet the basic nutrient requirements of rapidly growing tea bushes [30]. Given these trade-offs, combining organic and inorganic fertilizers offers a potential solution. Supporting this, a meta-analysis by Zhang et al. [31] which integrated 211 datasets from 35 studies demonstrated that various organic–inorganic fertilizer combinations promoted tea bush growth. On average, these combinations increased the tea water extract content by 3.73% and free amino acid content by 12.9%, further validating the advantages of COF. It is evident that the combination of organic and chemical fertilizers is conducive to improving the content of most quality indices of tea leaves, which has been supported by many studies and confirmed by the results of this study.

Volatile components are the key indices for evaluating tea aroma quality, and their composition and content directly affect aroma characteristics, quality grades, and stylistic features of tea [32]. Changes in fertilization significantly altered the composition and content of volatile components, thus affecting tea aroma characteristics [16]. Chen et al. [33] found that chemical fertilizers were beneficial in increasing the content of four key volatile components in tea leaves, but long-term use would reduce their content. The effects of chemical and organic fertilizer treatments on the aroma characteristics of green tea were studied by Huang et al. [34], who found that differences in fertilization significantly affected the content of six key volatile components in tea bush leaves. In the present study, it was found that the composition and content of volatile components in tea bush leaves altered significantly under different fertilizer treatments, especially the 11 characteristic metabolites of which 4 characteristic metabolites were highest in content under CF treatment, 8 were highest under OF treatment, and only 1 was highest under COF treatment. It is clear that fertilization treatments could affect the volatile components and their contents in tea leaves, and significant differences existed in the effects of different fertilizer treatments on tea bush leaves, which in turn might affect the aroma characteristics of tea leaves. Different types of volatile components exhibit different odor characteristics, thus forming special aroma qualities of tea [35]. Characteristic volatile components are particularly critical in forming tea’s special odor characteristics [36]. In this study, odor characteristics of the 11 characteristic metabolites obtained revealed that they mainly presented three types of odor characteristics, such as woody, floral, and green. The intensity analysis of odor characteristics showed that OF treatment was beneficial to enhance the intensity of odor characteristics such as woody and green, CF treatment was beneficial to enhance the intensity of flower, and the effect of COF treatment on the intensity of the three odor characteristics was intermediate. Yang et al. [37] analyzed the effects of different organic and chemical fertilizers on the flavor of Shuchazao (*Camellia sinensis*). In this, nine different volatile components were screened, of which the organic fertilizer treatment significantly enhanced the content of five volatile components, which were conducive to the enhancement of green and fruity odor characteristics. Qiu et al. [38] investigated the effect of chemical fertilizer on the intensity of volatile components in Lingtou Dancong (*Camellia sinensis*) and found that chemical fertilizers could significantly increase the content of five key volatile components and enhance the floral odor characteristics of tea. It is clear that some differences exist in the effects on different tea varieties regarding tea aroma quality under different fertilization treatments. And the results of this study indicated that the application of chemical fertilizers or organic fertilizers alone were conducive with enhancing the intensity of individual odor characteristics of tea, whilst at the same time also reducing the intensity of other odor characteristics. Meanwhile, chemical fertilizers and organic fertilizers were beneficial to the balance of these different odor characteristics after matching the application.

## 5. Conclusions

**Figure 6:**
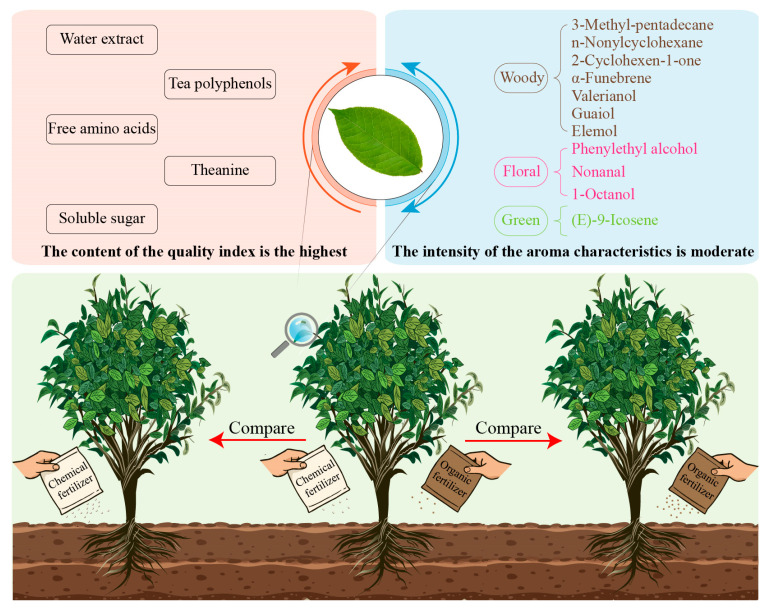
Effects of different fertilization treatments on tea quality.

In this study, different fertilizer treatments were used to treat tea bushes and their effects on quality indices and volatile components were analyzed. The results showed (Figure 6) that COF treatment was more beneficial in enhancing most of the quality indices of tea bush leaves, except caffeine and total flavonoids contents, compared to CF and OF. Significant alterations in the composition and content of volatile components, especially the 11 characteristic components, were observed in tea bush leaves after different fertilizer treatments. Analysis of odor characteristic intensities showed that OF treatment was beneficial to enhancing the intensity of the odor characteristics such as woody and green of tea bush leaves, CF treatment was beneficial to enhancing the intensity of floral, and the effect of COF treatment on the intensities of the three odor characteristics was intermediate. It is clear that different fertilizer treatments significantly affected the content of quality indices and tea aroma quality, and the use of chemical fertilizers or organic fertilizers alone was beneficial to enhance the intensities of individual special odor characteristics in tea leaves. This fertilization pattern can be used to strengthen the special aroma quality of tea cultivation and management. From the perspective of the overall improvement of tea quality, the combined application of chemical fertilizers and organic fertilizers was more conducive to enhancing the overall quality of tea. However, how to regulate the proportion of chemical fertilizers and organic fertilizers to achieve the best effect of tea quality enhancement requires in-depth research. Secondly, field fertilization treatment is influenced by various environmental factors and may have certain differences. Therefore, multi-year and multi-point experiments are still needed in the future to obtain more systematic and complete results. The present study offers a key basis for the application of fertilizer to regulate the growth of tea bushes and tea quality.

## Data Availability

The original contributions presented in this study are included in the article/Supplementary Materials. Further inquiries can be directed to the corresponding authors.

## Supplementary Materials

The following supporting information can be downloaded at: https://www.mdpi.com/article/10.3390/foods14183194/s1. Table S1. Data on volatile components in tea bush leaves after different fertilizer treatments.
